# Reversibility of calcinosis in anti-NXP2-positive refractory dermatomyositis treated with TNF-α blockade: a brief report

**DOI:** 10.3389/fmed.2026.1795210

**Published:** 2026-05-07

**Authors:** Hui Huang, Haiguo Yu, Zhidan Fan, Yihong Guo, Huihui Ma, Na Huang, Le Ma

**Affiliations:** Department of Rheumatology and Immunology, Children’s Hospital of Nanjing Medical University, Nanjing, China

**Keywords:** anti-NXP2 antibody, calcinosis cutis, dermatomyositis, steroid-sparing therapy, TNF-α inhibitors

## Abstract

**Background:**

Calcinosis cutis is one of the most refractory complications of dermatomyositis (DM), particularly in patients with anti-nuclear matrix protein 2 (NXP2) autoantibodies. Whether tumor necrosis factor-alpha (TNF-α) blockade is associated with regression of established calcinosis remains uncertain.

**Methods:**

We conducted a retrospective, single-center study of nine patients with refractory dermatomyositis or juvenile dermatomyositis treated with TNF-α inhibitors (infliximab or adalimumab). Myositis-specific autoantibodies, longitudinal muscle strength (MMT8, CMAS), glucocorticoid exposure, and serial imaging of calcinosis were evaluated. Calcinosis change was analyzed as an exploratory endpoint.

**Results:**

Three patients were anti-NXP2 positive, and six were negative for tested myositis-specific autoantibodies. TNF-α blockade was associated with sustained clinical improvement and a marked steroid-sparing effect, with median daily prednisolone dose decreasing from 40 mg at baseline to 5 mg at 12 months. Radiographic regression of calcinosis, defined *a priori* as a ≥20% reduction in maximal lesion diameter on serial imaging, was observed in 2 of 3 anti-NXP2-positive patients after prolonged treatment (12–20 months): notably, calcinosis was absent at baseline in all anti-NXP2-negative patients, precluding assessment of regression in this subgroup. One patient with pre-existing interstitial lung abnormalities experienced pulmonary deterioration, leading to treatment discontinuation.

**Conclusion:**

In this small retrospective cohort, prolonged TNF-α blockade was associated with radiographic regression of calcinosis in a subset of anti-NXP2-positive patients, while calcinosis was absent at baseline in antibody-negative patients. These findings generate a hypothesis that antibody-defined subgroups may differ in calcinosis responsiveness and warrant prospective validation.

## Introduction

Dermatomyositis (DM) is a heterogeneous autoimmune inflammatory myopathy characterized by proximal muscle weakness, typical cutaneous manifestations, and variable systemic involvement. Despite advances in immunosuppressive and biologic therapies, a proportion of patients—particularly those with juvenile-onset disease—develop refractory disease with irreversible tissue damage (1, 2). Calcinosis cutis is among the most disabling and treatment-resistant complications of DM, especially in juvenile dermatomyositis (JDM) (3). Once established, calcinosis is traditionally considered largely irreversible and is associated with pain, infection, functional limitation, and reduced quality of life (4). Accumulating evidence links calcinosis to anti-nuclear matrix protein 2 (NXP2) autoantibodies, which define a severe, damage-prone disease phenotype (5–7).

Tumor necrosis factor-alpha (TNF-α) plays a central role in inflammatory myopathies and the maintenance of a pro-calcific inflammatory microenvironment (8, 9). Whether sustained TNF-α blockade can induce regression of established calcinosis, particularly in antibody-defined subgroups, remains unclear. We therefore evaluated longitudinal clinical and radiographic outcomes of TNF-α inhibition in refractory DM, with a focused exploratory analysis according to anti-NXP2 antibody status.

## Methods

### Study design and patients

This retrospective, single-center study was approved by the Institutional Ethics Committee of the Children’s Hospital of Nanjing Medical University. Written informed consent was obtained from the legal guardians of all participants. Patients with definite or probable dermatomyositis or juvenile dermatomyositis diagnosed according to Bohan and Peter criteria were eligible. Refractory disease was defined as persistent moderate-to-severe activity despite at least 6 months of high-dose glucocorticoids in combination with two or more conventional immunosuppressive agents. Prior immunosuppressive therapies included methotrexate, mycophenolate mofetil, cyclosporine, tacrolimus, intravenous immunoglobulin, and cyclophosphamide, administered in various combinations before initiation of TNF-α blockade.

### Ethics approval and consent to participate

Ethics approval was obtained from the Institutional Ethics Committee of the Children’s Hospital of Nanjing Medical University (Approval No. 202012106-1). The study was conducted in accordance with the principles of the Declaration of Helsinki. Written informed consent to participate was obtained from the legal guardians of all participants prior to enrollment.

### Treatment

All patients received TNF-α inhibitors as add-on therapy, including infliximab (6 mg/kg every 4 weeks) or adalimumab (24 mg/m^2^ every other week), according to clinician judgment and patient characteristics.

### Assessments

Myositis-specific autoantibodies tested in our cohort included anti-NXP2, anti-Mi-2, anti-MDA5, anti-TIF1-γ, anti-SAE1, anti-SRP, anti-HMGCR, and anti-aminoacyl-tRNA synthetase antibodies (including anti-Jo-1, anti-PL-7, and anti-PL-12), which were measured using standardized immunoassays routinely available at our center. Clinical outcomes included Manual Muscle Testing-8 (MMT8), Childhood Myositis Assessment Scale (CMAS), and daily prednisolone dose, assessed at baseline and during follow-up.

Calcinosis was evaluated using serial radiography, with the same imaging modality and anatomical region used whenever possible. Radiographic change was predefined as a ≥ 20% reduction in the maximal diameter of the largest calcific deposit measured electronically. The same lesion was consistently selected for longitudinal measurement across serial imaging. The 20% threshold was predefined pragmatically to capture meaningful directional change rather than definitive regression. Two senior radiologists, blinded to clinical data and treatment phase, independently reviewed all images; discrepancies were resolved by consensus. Calcinosis outcomes were considered exploratory and hypothesis-generating.

### Statistical analysis

Continuous variables are reported as medians with interquartile ranges. Given the small sample size and incomplete follow-up at later time points, analyses of muscle strength were descriptive. Changes in glucocorticoid dose over time were assessed using non-parametric paired tests where appropriate. A two-sided *p* value <0.05 was considered statistically significant for glucocorticoid analyses only.

## Results

### Patient characteristics

Nine patients (median age 7.8 years) were included. Three patients were anti-NXP2 positive, and six were negative for tested myositis-specific autoantibodies. All patients had active disease despite combination immunosuppressive therapy prior to TNF-α inhibitor initiation (Table 1).

**Table 1:** Baseline characteristics and key outcomes of patients with refractory dermatomyositis treated with TNF-α inhibitors.

Characteristic/Outcome	Overall (*N* = 9)	NXP2-positive (*n* = 3)	NXP2-negative (*n* = 6)
Baseline characteristics			
Age at TNF-α initiation, years, median (IQR)	7.8 (6.4–10.2)	7.5 (6.8–9.1)	8.1 (6.2–10.8)
Sex, female, *n* (%)	5 (55.6)	2 (66.7)	3 (50.0)
Pediatric-onset DM, *n* (%)	8 (88.9)	3 (100)	5 (83.3)
Disease duration before TNF-α therapy, months, median (IQR)	14 (10–22)	16 (12–24)	13 (9–21)
Calcinosis at baseline, *n* (%)	3 (33.3)	3 (100)	0 (0)
Interstitial lung abnormalities at baseline, *n* (%)	1 (11.1)	0 (0)	1 (16.7)
Prior immunosuppressive agents ≥2, *n* (%)	9 (100)	3 (100)	6 (100)
Baseline disease activity			
MMT8 score, median (IQR)	140 (40–150)	135 (40–140)	150 (140–150)
Daily prednisolone dose, mg/day, median (IQR)	40 (25–50)	40 (40–60)	35 (25–45)
Key outcomes during follow-up			
Improvement in MMT8 at 12 months, *n* (%)	8/9 (88.9)	3/3 (100)	5/6 (83.3)
Prednisolone dose at 12 months, mg/day, median (IQR)	5.0 (5.0–6.9)	5.0 (5.0–6.3)	5.0 (5.0–7.5)
Steroid-sparing effect^*^, *n* (%)	9 (100)	3 (100)	6 (100)
Radiographic regression of calcinosis, *n*/*N* (%)	2/3 (66.7)	2/3 (66.7)	0/6 (0)
Pulmonary adverse events, *n* (%)	1 (11.1)	0 (0)	1 (16.7)

DM, dermatomyositis; MMT8, Manual Muscle Testing-8; IQR, interquartile range.

Steroid-sparing effect was defined as a sustained reduction in daily prednisolone dose compared with baseline.

Radiographic regression of calcinosis was defined as a ≥20% reduction in the maximal diameter of the largest calcific deposit on serial imaging.

NXP2-negative indicates negative for tested myositis-specific autoantibodies.

*Steroid-sparing effect, defined as a sustained reduction in daily prednisolone dose compared with baseline.

### Clinical response and steroid-sparing effect

**Figure 1:**
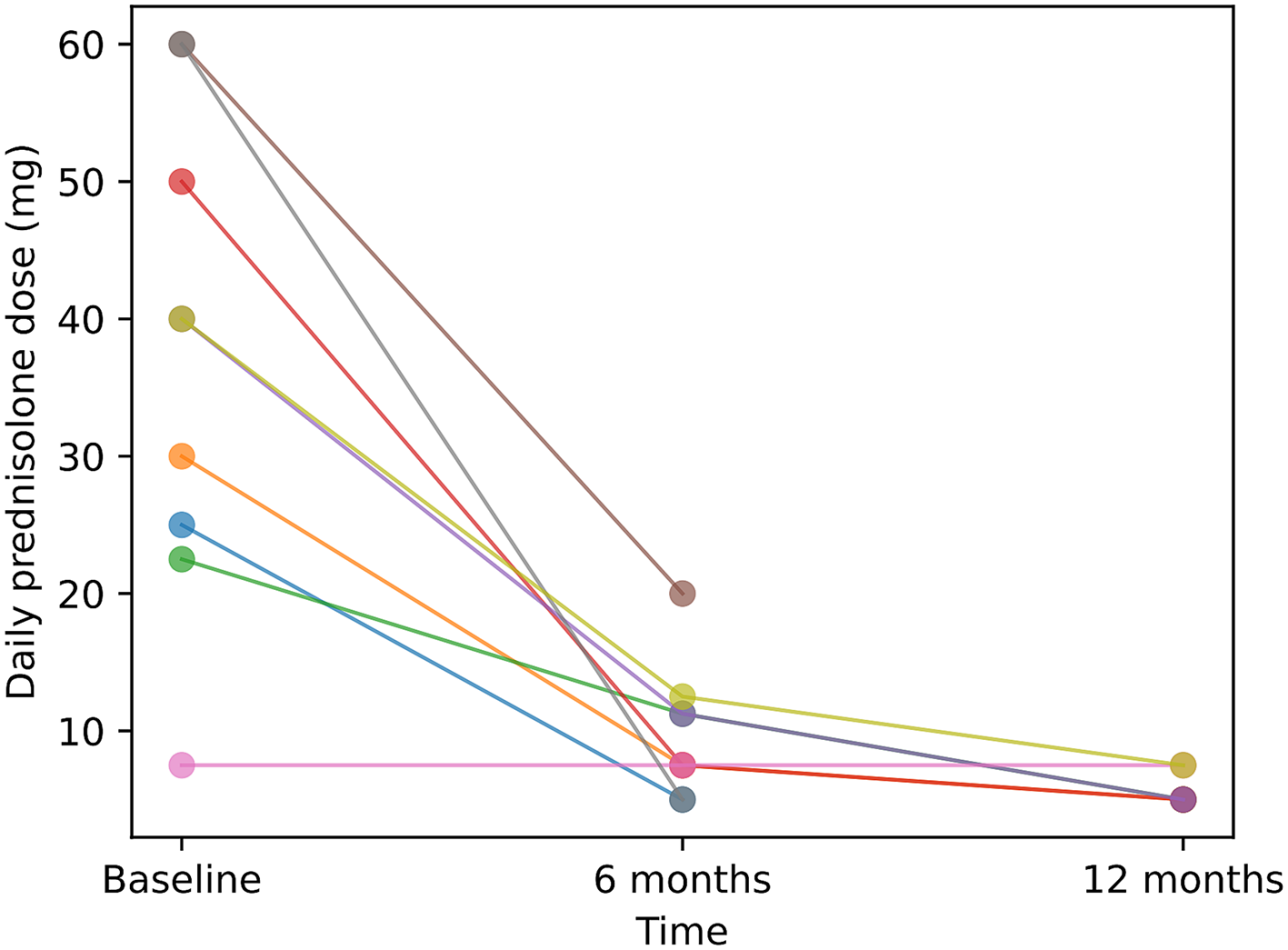
Individual trajectories of daily prednisolone dose following TNF-α blockade. Each line represents one patient. A consistent reduction in daily prednisolone dose was observed over time in most patients, illustrating a steroid-sparing effect. No statistical comparisons were performed.

Following TNF-α blockade, most patients demonstrated sustained improvement in muscle strength over follow-up. One patient with severe baseline weakness (MMT8 = 40) achieved near-complete functional recovery within 12 months. Median daily prednisolone dose decreased from 40 mg at baseline to 11.25 mg at 6 months and further to 5 mg at 12 months (Figure 1), reflecting a substantial steroid-sparing effect. Because median MMT8 values were unchanged across time points, formal statistical testing for muscle strength was not emphasized.

### Calcinosis outcomes

**Figure 2:**
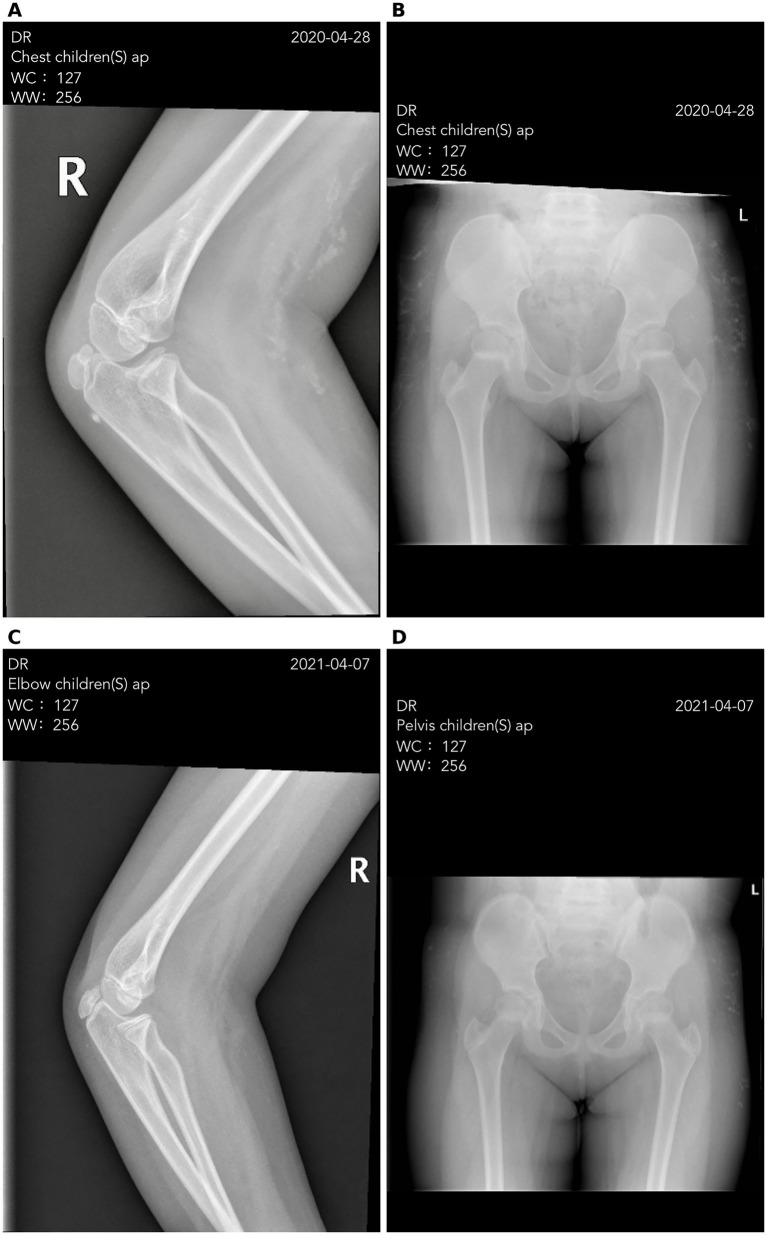
Radiographic regression of calcinosis following TNF-α blockade in an NXP2-positive patient. **(A,B)** Baseline radiographs (April 28, 2020) show periarticular soft-tissue calcific deposits (arrows). **(C,D)** Follow-up radiographs after prolonged TNF-α inhibition (April 7, 2021) demonstrate reduced maximal diameter and radiographic density of corresponding deposits (arrows). Radiographic improvement was defined as ≥20% reduction in the maximal diameter of the largest calcific deposit and was independently assessed by two radiologists blinded to clinical outcomes.

Radiographic regression of calcinosis was observed exclusively among anti-NXP2-positive patients. Two of three anti-NXP2-positive patients showed a ≥20% reduction in maximal calcific lesion diameter after prolonged TNF-α inhibition (12–20 months) (Figure 2). In contrast, calcinosis was present at baseline exclusively in anti-NXP2-positive patients. No calcinosis was observed in anti-NXP2-negative patients at baseline or during follow-up. These findings suggest a potential antibody-defined difference in calcinosis responsiveness. Given the small sample size and exploratory nature of this study, outcomes were summarized at the cohort level and were not analyzed according to individual TNF-α agents.

### Safety

TNF-α inhibitors were generally well tolerated. One patient with pre-existing interstitial lung abnormalities experienced pulmonary deterioration after treatment initiation, prompting discontinuation. No serious infections or deaths occurred.

## Discussion

In this Brief Report, prolonged TNF-α blockade was associated with radiographic regression of calcinosis in a subset of anti-NXP2-positive patients with refractory dermatomyositis, whereas no calcinosis was observed in antibody-negative patients. The delayed onset of regression after extended treatment contrasts with the traditional view of calcinosis as an irreversible form of disease damage (3, 4).

The antibody-restricted signal observed is biologically plausible, as anti-NXP2 autoantibodies are consistently associated with severe, damage-prone DM phenotypes, including extensive calcinosis (5–7). Sustained TNF-α-driven inflammation and macrophage activation are thought to contribute to a pro-calcific tissue microenvironment, providing a potential mechanistic rationale for the time-dependent effects observed with TNF-α inhibition (8, 9).

Despite increasing use of biologic therapies in DM, effective treatments for calcinosis remain limited and largely exploratory (10–12). In this context, our findings suggest that antibody-defined stratification may help identify patients more likely to benefit from targeted interventions. Safety considerations remain important; pulmonary deterioration observed in one patient underscores the need for careful selection and monitoring when using TNF-α inhibitors (13).

The limitations of this study include its retrospective design, small sample size, and exploratory assessment of calcinosis. Accordingly, these findings should be interpreted as hypothesis-generating and require prospective validation.

## Conclusion

In summary, this Brief Report suggests that prolonged TNF-α blockade may be associated with radiographic regression of calcinosis in a subset of anti-NXP2-positive patients with refractory dermatomyositis, while calcinosis was absent at baseline in antibody-negative patients. These findings should be interpreted as hypothesis-generating and underscore the need for prospective studies to clarify the role of antibody-defined stratification in the management of calcinosis.

## Data Availability

The raw data supporting the conclusions of this article will be made available by the authors, without undue reservation.
